# Adaptation to Long-Term Home Non-Invasive Ventilation for People with Chronic Hypercapnic Respiratory Failure: A Qualitative Study

**DOI:** 10.3390/nursrep15050176

**Published:** 2025-05-20

**Authors:** Nur Zahrah Yuko Yacob Hussain, Norasyikin Hassan, Hang Siang Wong, Yingjuan Mok, Piyanee Klainin-Yobas

**Affiliations:** 1 Division of Nursing, Changi General Hospital, Singapore 529889, Singapore; 2 Alice Lee Centre for Nursing Studies Yong Loo Lin School of Medicine, National University of Singapore, Singapore 117597, Singapore; 3 Department of Sleep Medicine, Surgery and Science, Changi General Hospital, Singapore 529889, Singapore; 4 Department of Respiratory and Critical Care Medicine, Changi General Hospital, Singapore 529889, Singapore

**Keywords:** hypercapnic respiratory failure, non-invasive ventilation, physiological adaptation, psychological adaptation, qualitative research

## Abstract

**Background/Objectives:** Home non-invasive ventilation use is the primary treatment for improving respiratory function in people with chronic hypercapnic respiratory failure. Non-invasive ventilation has also been studied to understand users’ perspectives. However, no studies have been conducted on how users adapt to non-invasive ventilation in their homes from the early phase of their diagnosis as a long-term utility. **Methods:** The study employed a descriptive qualitative design guided by Roy’s adaptation model. A purposive sample was used. People with chronic hypercapnic respiratory failure who had used NIV at home for a minimum of six months would be eligible. They were interviewed at a sleep and assisted ventilation centre. Their interviews were audio recorded before proceeding with transcription. Each transcript was thematically analysed. **Results:** Twenty participants were included in the study, from which six themes emerged. They experienced a common transition, from denying the need for non-invasive ventilation to integrating them into their daily lives at home. Throughout this process, they had emotional turmoil, faced difficulties in keeping their masks on, and improved sleep quality. They also adjusted their social interactions before fully accepting the use of non-invasive ventilation. Their coping strategies in their role functions at home and social interaction were also narrated. Their family members were pivotal in their adaptation period. **Conclusions**: Gaining insight into individuals’ adaptation experiences can facilitate early identification of potential challenges faced by new users of non-invasive ventilation. This study calls for healthcare professionals to assess users’ understanding of long-term commitment and their living conditions early for a successful NIV adaptation.

## 1. Introduction

Chronic hypercapnic respiratory failure (CHRF) resulting from chronic obstructive pulmonary disease (COPD), obesity hypoventilation syndrome (OHS), or neuromuscular diseases (NMDs) is characterised by hypercapnic symptoms, including headaches, daytime sleepiness, and impaired concentration [1]. CHRF has been associated with significant morbidity, mortality, and frequent hospitalisation [2,3]. Non-invasive ventilation (NIV) has been the early intervention in treating people with CHRF and has become the first-choice modality for achieving their optimal oxygenation and ventilatory parameters [4,5]. It has been studied extensively for its effectiveness in physiological parameters, its evaluation of survival outcomes, hospital readmissions, and health costs [6,7,8]. Longitudinal studies measuring the effects of home NIV on health-related quality of life (HRQoL) using the SF-36 questionnaire on people with CHRF and its subgroups (COPD, OHS, NMDs) have demonstrated improved HRQoL over 2 years [9,10]. However, studies are reporting that they also suffer from anxiety, depression, and other cognitive impairments that interact with the clinical condition, impacting their disease burden [11,12,13]. These conditions affect their long-term treatment and compliance, which informs a different scope of inquiry in managing their illness and personal experiences.

Some studies undertook a qualitative approach to understanding their illness by exploring the NIV user experience [14,15,16,17,18]. However, there is no qualitative evidence on how individuals adapt to using NIV at home during the early stages of their diagnosis. This phase typically involves hospital monitoring and frequent outpatient visits to respiratory physicians to help integrate NIV into their daily routines [7]. These episodes of their illness require an adaptation in their personal lives, acclimatisation to the ventilator, and plausibly accommodation in their homes. Living with a chronic illness can lead to a significant bio-psycho-social disruption. In the early stages of managing the chronic hypercapnic symptoms, the symptoms often interfere with their mental health and lifestyles. Over time, this disruption becomes a persistent aspect of the individual’s condition, which requires them to adapt to it to continue living. The empirical evidence above agrees with the core element of Callista Roy’s adaptation model, proposing that humans constantly interact with their environment to regain the unity and integrity of their health [19]. Learning and recognising the adaptation phase of their illness is essential for healthcare professionals to understand and address their patients’ concerns about managing their chronic symptoms and using their NIV. The study aimed to interpret the experiences of people with CHRF living with NIV at home, guided by Roy’s adaptation model.

### Theoretical Framework

Roy’s adaptation model stems from the concept that humans continuously interact with their environment (stimuli) during health adaptation. The stimuli are termed as focal, contextual, and residual [20]. Focal stimuli are what the person faces the most. Contextual stimuli contribute to the person’s experience and learning, which may affect how the person deals with the focal stimuli [20,21]. Residual stimuli are indeterminate factors in the human environment that are not central to the primary behaviour [22,23]. The ability to adapt to the stimuli is essential. The model describes that a person requires an adaptive system manifested through a coping mechanism. It presents four adaptive modes: self-concept, physiological, role function, and interdependence [19]. These adaptive modes informed the scope of the interview questions to gather relevant narratives. They guided the study team in answering the research question, *‘What is the adaptation experience of people with chronic hypercapnic respiratory failure living with a long-term non-invasive ventilator at home?’*

## 2. Materials and Methods

The study employed a qualitative design with an inductive approach to describe the experiences of people with CHRF. They depend on a home NIV to maintain their oxygenation and ventilatory function. The chosen method allows a naturalistic inquiry to explore how people interpret their life experiences in ordinary settings [24].

### 2.1. Participants and Setting

Purposive sampling was used to recruit people with the stated experiences. The study recruitment was conducted at a sleep and assisted ventilation centre of a medical institution in Singapore. People with CHRF from COPD, OHS, and NMDs who had used NIV at home for a minimum of six months were recruited. People who were newly diagnosed with CHRF and new NIV users were excluded.

### 2.2. Data Collection

The data collection was conducted from August to September 2023. Potential participants were invited to participate in the study at the end of their medical outpatient consultations with their respiratory physicians at the centre. They were brought to a room where the principal investigator and co-investigator introduced the study. Potential participants were given a Participant Information Sheet to explain the study aims and their roles before making an informed decision. They were given time to read the information sheet at the sleep and assisted ventilation centre. Their queries were answered by the principal investigator and the co-investigator before written consent was obtained.

To ensure privacy, a one-time, audio-recorded, face-to-face interview was conducted in a room away from the centre’s consultation rooms. Twenty participants were recruited. One potential participant declined to participate because the individual did not wish to be recorded. Four participants who were in wheelchairs came with their family members or caregivers to the room. If the participants were comfortable being interviewed in their presence, permission was sought from them. Other participants came alone to participate in the study. The interview questions guided by Roy’s four adaptive modes were used during the interview session. Each interview lasted for 25 min. Repeated contents of the participants’ narratives occurred at the 15th interview. Five more interviews were conducted until there was little or no new information, suggesting data saturation after concurrence with the principal investigator [25].

### 2.3. Ethical Considerations

This study received ethical approval from the Hospital Institutional Review Board and adhered to the ethical principles outlined in the Belmont Report [26]. Study participation was voluntary. Anonymity and confidentiality were ascertained using pseudonyms and secured data storage methods.

### 2.4. Analysis

Braun and Clarke’s six-step thematic analysis was employed to identify and elicit semantic patterns from the transcripts [27]. The initial process began with a reflective debriefing by the principal investigator and the interviewer after each interview to gather thoughts about the participants’ stories. This was followed by transcription of the audio recordings after each interview. The transcripts were first read to obtain a sense of their stories. Subsequent repeated reading was purposefully performed to immerse into the participants’ experiences to elicit semantic codes. All the codes were transferred to a Microsoft Excel sheet to keep track of the iterative changes along with the participants’ line-by-line statements [28]. At this point, the recursive process of returning to the original transcripts was also performed to ascertain meaningful interpretation before generating candidate themes. The themes were shared with the study team, allowing a reflexive revision process before finalising them with the participants’ quotes from the interview.

### 2.5. Trustworthiness

The trustworthiness criteria established by Lincoln and Guba were used to evaluate the study’s rigour [29]. Credibility, dependability, transferability, and confirmability were incorporated into the research process. To validate the truth of the findings, the study team continued to engage with the participants during the interview. This clarified any nuances of their adaptation to their NIV and their feelings about their illness for credibility. The study team maintained an audit trail by detailing the research process and interview transcripts, keeping records of the data analysis for dependability [30]. For transferability, purposive sampling was employed to select participants with relevant experiences to be studied. Participants’ demographics and the study context were documented for potential applicability in other contexts. Confirmability was established by incorporating the opinions of the entire study team regarding the thematic findings and clinical conditions. Verbal quotes from the participants were also integrated into the thematic findings.

## 3. Results

Twenty participants completed the interview. Most were female (*n* = 12). Many of the participants were 65 years of age and older. The ethnic group was diverse, with many Chinese participants. The participants’ demographics are presented in Table 1.

All participants were diagnosed with chronic hypercapnic respiratory failure, with many caused by OHS and NMDs, and were using NIV at home at night for a minimum of 6 months to more than one year. Five participants shared that they used oxygen therapy as a supplementary measure to manage shortness of breath during daytime. The participants’ clinical information is presented in Table 2.

The thematic analysis revealed six themes detailing their adaptation to NIV at home and how it integrated into their lives. The sub-categories, categories, and themes are outlined in Table 3 and Supplementary Materials. Each theme is supported by participant quotations, identified by their pseudonyms.

Through the in-depth interviews, participants shared a common transition—from NIV denial to life adjustments and NIV integration. The six themes encompass their emotional turmoil, changes in sleep quality, challenges in maintaining an NIV mask, social adjustments, and making NIV part of their lives. Each theme highlights specific aspects of the participants’ adaptation to NIV.

### 3.1. Theme 1: Forced Acceptance to Use NIV

In their early adaptation period, the participants reported a complex journey of acceptance towards home NIV, marked by excitement and disappointment. At first encounter, they expressed ambivalence, balancing the prospect of a novel treatment with the awareness of its lifelong implications. Repeated hospital admissions due to hypercapnia were the impetus for them to accept NIV. They expressed a dual response to using NIV; some were curious about its benefits yet reluctant to accept the long-term obligation.

*“It is something new that you try, you see. Of course, you feel excited.”*

(*Lily*)

*“I was disappointed because I (must) use all these things (mask and ventilator) for my whole life. You know, it will be quite a long time. Yes, but then, because of my sickness, I have no choice.”*

(*Jennifer*)

Many participants initially viewed NIV as a trial to assess its effectiveness in improving oxygenation levels. This evaluation period allowed them to recalibrate their expectations and become familiar with the ventilator interface while integrating its use into their daily routines. Over time, they used NIV more consistently to manage their respiratory condition, eventually regarding it as a vital component of their treatment plan.

*“Sometimes I (was) fed up. I want(ed) to throw (NIV) away. I don’t want to do. Then after a while, I think, no. It’s for my own good.”*

(*Evelyn*)

### 3.2. Theme 2: Experiencing Symptoms Relief and Side Effects

During the NIV trial, the participants attempted to find the proper placement of their masks on their faces, with some having to return to the sleep and assisted ventilation centre to obtain a suitable mask fit. Participant Anne had “nightmares” with the illusion of something pressing on her face at night. Another participant, Khris, perceived that the mask could suffocate him. During such difficult times, their negative perceptions of NIV were soon overturned when they started to experience relief from morning headaches immediately after full use of the NIV the night before. Other common CHRF symptoms, like daytime sleepiness and poor concentration, were alleviated significantly. Their improved sleep contributed to better concentration and reduced daytime sleepiness, facilitating greater engagement in their day tasks.

*“When I wake up, I don’t have a headache anymore, and I don’t have that very frustrated feeling when I wake up. You know, it’s easier to wake up with a better mood.”*

(*Anne*)

*“After using it, after some time, I really feel that it makes a difference in the ability to concentrate. It lasts longer during the days. It has a significant impact when I reflect (on) it.”*

(*Victor*)

Many participants expressed increased physical endurance, including walking longer distances and engaging in daily activities with reduced episodes of breathlessness. For Yayah, she lost weight from “120 kg to 94 kg” from being active since using NIV. At the same time, they reported side effects, such as dry mouth, gum pain, and medical-device-related pressure injuries, which introduced additional complexities in adapting to the NIV mask.

*“Every night I must (put) a plaster because of (the) pain. Then, here also must plaster (points to cheeks and chin), here also must plaster, nose.”*

(*Mary*)

*“Initially (it) was quite uncomfortable because (the) next morning my throat is very, very dry like having a sore throat, dry throat.”*

(*William*)

### 3.3. Theme 3: Learning to Maintain the NIV Mask

This theme emerged as the participants began their journey to manage their masks independently or with the assistance of caregivers. During this phase, they learned about the technical adjustments necessary for their masks in consultation with their respiratory physicians and the costs of purchasing new masks and accessories.

Achieving a secure fit and managing air leaks were common challenges, necessitating ongoing mask fitting and size adjustments. Regular appointments with their respiratory physicians provided valuable support, allowing participants to seek help for fitting issues when necessary. The participants and their families also learned to obtain supplies from the NIV providers when mask accessories were damaged or needed replacement.

*“I frequently see the doctors to find the suitable size. We had the sleep study. They tested the pressure, the mask size, and the strap.”*

(*Muhammad*)

Initially, their understanding of proper mask maintenance was limited. Some shared stories of disinfecting their masks with alcohol wipes and hanging them near a window under direct sunlight, unaware that such practices could potentially damage their masks. Maria described how she used to clean her mask this way:

*“I used Mama Lemon (dishwashing liquid) to wash every 2 weeks.”*

Reading the ventilator manual and receiving guidance from the NIV providers taught them effective cleaning methods. They also recognised that failing to follow the instructions could damage the mask, necessitating a new purchase. As a result, the participants became aware of the importance of maintaining a mask to avoid additional expenses for replacing masks and accessories. Some participants added that the ventilator was expensive and required medical social assistance to purchase it.

### 3.4. Theme 4: Incorporating NIV into the Home Environment

Their experiences with their masks depict a daily affair with care and caution to achieve uninterrupted sleep. As they became more accustomed to their nightly NIV routine, they shared modifications made to their home environment in the interviews. The participants typically positioned the ventilator on a bedside table for easy access or close to an electric wall socket to charge the ventilator. Two participants wore adult diapers during sleep to avoid going to the toilet. Another participant even redesigned a rack with wheels for her NIV so she could easily walk to the bathroom without removing her mask at night.

*“I put it on the rack-like trolley and then bring the whole thing to the toilet door. Then I go and do (urinate).”*

(*Mary*)

The participants also became accustomed to the noise generated by the device and the air leakage sounds, occasionally disrupting their sleep. During this period, people who lived with them joined the adaptation phase by changing their sleep location and getting woken up by the alarm sounds. These people were their family members or caregivers who adjusted their night routines, with some choosing to sleep separately but near the participant’s bedroom to provide comfort and assistance when needed. These narrations were commonly shared by the families of participants with NMDs or those who were less independent.

*“It’s quite impossible to sleep in the same room, so my mom sleeps in a separate room. Yeah, and I’ll be at the hall for the night. So, I think that’s an arrangement for family if you need to take note of that.”*

(*Tee’s son*)

*“I hired a domestic helper. Her job is solely to take care of my mother…She sleeps in the same room.”*

(*Fatimah’s son*)

Participants who were partially or fully dependent on their daily care mainly relied on their family members for the NIV operation and maintenance. Fatimah’s son expressed anxiety on one occasion when the machine needed repair, anticipating the likelihood that his mother would become breathless and need medical attention. The perspectives from the participants’ families informed that the integration of NIV in their homes impacts the participants’ lives and the people living with them. They became supporters for troubleshooting alarms or purchasing a new mask. It demonstrates their care and concern for their family members with CHRF since NIV is in their homes. Some participants expressed gratitude to their families for adapting to NIV together.

*“He will try to comfort me when I get frustrated (with my illness). He would say, “Lyn, bear with it.” He is a very good husband [Evelyn cries].”*

(*Evelyn*)

### 3.5. Theme 5: Readjusting Travelling Activities

Owning an NIV required the participants to modify their social routines and interactions. Many participants perceived it as “troublesome”, with some opting to avoid travel entirely and others choosing to stay home most of the time. This resulted in their family visiting them at home instead. Some participants experienced temporary social disconnection from their work peers due to extended sick leave. When travel was unavoidable, there were mixed responses about travelling with their NIV machines.

*“When we have to travel, we have to travel with oxygen tanks and everything. So that’s a troublesome part…The machine is big.”*

(*Tee’s son*)

*“I’m very worried if I bring (it), I don’t know whether I will damage the machine.”*

(*Anne*)

Other participants shared that their experiences travelling with their NIV were positive and did not deter them from going overseas.

*“When I went to Australia, I brought it…This thing (NIV) is not that heavy.”*

(*Yayah*)

This aspect of their adaptation clearly demonstrated a strong desire to return to everyday lifestyles, particularly in relation to travelling. They actively assessed how bringing an NIV unit would affect their travel plans. Responses to these decisions were diverse, leading to various effective coping strategies as they adjusted their lifestyles. While some chose to avoid travel entirely, others confidently incorporated travel into their plans. Moreover, this deliberation fosters an early acceptance of the NIV as an essential factor in their decision-making for travel and outdoor activities.

### 3.6. Theme 6: NIV as Part of Their Lives

This theme was derived inductively as the participants shared that putting on their NIV masks became automated and perceived the task as “simple,” as described by Anne. The NIV has become like a ‘companion’ to live with their chronic illness. Jennifer described her NIV this way:

*“It is now my “BFF” (best friend forever). Cannot live without it.”*

(*Jennifer*)

They also recognised the risks of reduced oxygen saturation and the need to maintain adequate oxygen levels. Reliance on the NIV led to anxiety, particularly when the mask was damaged or the ventilator malfunctioned.

*“Without the machine (NIV), I will get breathless when I walk…when the mask has problems, consult the doctor and change for a new one.”*

(*Ah Hwee*)

These verbatim quotes highlighted how their attitudes towards their home NIV have changed, from a medical device that they resented to an essential item that they relied on to manage their chronic conditions. This shift also led them to take more responsibility for their respiratory care by recognising their trigger symptoms, identifying when their mask needed adjustment, and when to seek medical help or consultation.

## 4. Discussion

This study delved into the experiences of people with CHRF living with home NIV, guided by Roy’s adaptive modes. The six themes highlight the participants’ journey to acclimatise, adjust, and accept home NIV. In terms of NIV acclimatisation, there is evidence in their narratives that they were compelled to accept the NIV by framing it as an essential item for their health and well-being, which aligns with Roy’s explanation of self-concept. Regarding their adjustment to NIV, there was a gradual shift demonstrating the dynamic process through which they shifted their self-concept and identity in response to a new challenge or experience, which validates Roy’s explanation of stimuli through discovery and learning [19]. The stimuli drove the participants to see the physiological benefits of NIV, including improved sleep quality and symptom relief. These experiences underscore the tangible health improvements supporting Roy’s physiological adaptation mode.

Our first theme addressed how participants managed the NIV mask. Many shared their ongoing struggles to become comfortable with the mask and overcome NIV mask-related pressure injuries, which are also widely reported by NIV users in other studies, while striving for comfort and adherence to treatment [31,32,33]. These difficulties have impacted their role as parents, spouses, or employees in the initial period. As a result, they often find it challenging to socialise or are required to take sick leave from work. The role function and interdependence of adapting to NIV demand significant coping efforts for them to return to their usual roles or even develop a new approach to accepting NIV while living with their chronic illness.

The second theme highlighted the relief from CHRF symptoms, increased physical endurance, and weight loss while still experiencing side effects associated with wearing the NIV. Our findings are similar to a those of a qualitative study in Australia [18]. Specifically, many participants described “mental juggling” between the benefits and burdens of NIV. On the one hand, they experienced dyspnoea relief, better sleep, and less anxiety. NIV was perceived as a tool for recovery, which gave them another chance in life. On the other hand, NIV initiation led to suffering and functional limitations. A grounded study in Denmark [15] presented how participants using NIV progressed through a three-phase behavioural pattern: Initiation (struggling with NIV), Transition (coping with discomfort) and Determination (satisfied or dissatisfied conditions). Healthcare professionals need to understand this pattern of behaviour and provide care accordingly.

Our findings provided novel insights into how participants became involved in “learning to maintain the NIV mask” and “incorporating NIV into the home environment”, as described in Themes 3 and 4. While they learned to maintain their NIV mask in good condition, this study observed their social adjustment to home NIV. Their social adjustment reveals a dual response: they may either become isolated by staying at home or adjust their social activities to accommodate the use of NIV. Social isolation has been associated with chronic illnesses [34,35,36], and living conditions significantly influence their preferred recreational activities [37]. This study adds that medical devices at home also serve as a precursor that affects their social preferences. Some research suggests that friends may provide more support than family in these situations [38]. Depending on their communities, peer support for individuals with chronic respiratory illnesses can enhance their coping strategies [39,40]. However, it is also essential to recognise that individuals who live alone and have limited access to medical and social support services due to transportation, utility infrastructure, and urbanisation may experience different adaptation challenges [7]. This underscores the varying stages of their ability to self-manage at home [41,42].

Our study also identified the roles of families in illness monitoring, psychological support, and financial assistance, alongside healthcare professionals’ informational support and medical advice, as factors in their long-term adaptation to home NIV. The findings support the initiative of home visits by community nursing services, which have been reported to benefit users and their families by providing NIV training and addressing their challenges at home [43]. Additionally, participation in a post-discharge program has been associated with lower mortality rates and fewer emergency visits [44].

Travelling with NIV represented a significant adjustment in their lifestyles, as articulated in Theme 5. The immediate concerns regarding the risks of potentially damaging the machine or the inconvenience associated with transporting it during travel heavily influenced their decision-making process. A notable aspect was the involvement of family members, who could also impact their travel decisions by assisting in the planning and transportation of the NIV along with the requisite accessories. A retrospective study conducted in Canada emphasises the scarcity of literature on travel experiences, as families are required to prepare for increased medical complexities and to seek medical consultation from their residences [45]. Irrespective of their travel choices, the participants started to recognise NIV as integral to their decision-making and financial planning processes, aligning with their perception of “NIV as part of their lives.” This notion conveys a sense of closeness, deeply embedded in their lives, as they endeavour to maintain their integrity in health [19]. Our findings not only highlight the importance of social factors in the utilisation of medical devices for managing chronic conditions but also illuminate personal experiences associated with adapting NIV in domestic settings. This evidence serves to guide healthcare professionals in promptly assessing the personal needs of potential users, their home environments, and in involving their families at an early stage, if necessary. Such an approach can foster a long-term commitment to effectively adapt and utilise home NIV.

### Study Strengths and Limitations

The study’s strengths were the diverse age of participants recruited and the similarity of participants’ narrations about overcoming their NIV challenges at home. Many participants have been using the NIV for more than 6 months, which provides a broad sharing of their experiences. Another study strength is the involvement of their family members in some of the interviews, which validated the participants’ adaptation to home NIV and how they adjusted their home activities together with the participants.

The study identified several limitations. While the recruitment process gathered a diverse demographic profile, many participants were aged 65 and older, unemployed, or retired. The older participants may have already experienced various life stages, making them more accustomed to managing health-related interventions and more likely to accept home NIV than younger adults. The latter group reportedly faces the risk of discrimination from peers, parents, and at the workplace in managing their chronic illness [46], which can impact their adaptation period. The study findings may not accurately represent the adaptation of young adults to NIV, suggesting the need for further research to explore their adaptation requirements and challenges in using NIV to manage their respiratory symptoms.

The other limitation was the unemployment status of most participants. Their struggles and the high cost of purchasing an NIV unit and accessories were evident in their narratives; however, few expressed how they overcame their financial problems, which could provide insights into how they manage their medical expenses during unemployment or retirement. The financial burden remains a key variable influencing individuals to reject or discontinue medical treatment [45]. This study recommends exploring their financial enablers, which could offer a new perspective on managing their chronic respiratory issues.

The study did not specify how long their adaptation phase lasted. However, it unveils valuable insights into their experiences that would have remained concealed without the opportunity to share them during the interview sessions.

## 5. Conclusions

Chronic hypercapnic respiratory failure has lifelong implications for those living with the condition. Understanding their experiences with adaptation can help identify potential challenges new users may encounter when using NIV at home. The findings of this study highlight the complexity of adapting to home NIV, revealing not only the physical adjustments required but also the psychological, social, and emotional challenges involved. By recognising these varied aspects of adaptation, healthcare professionals can shift beyond the technical aspects of treatment towards a more holistic, person-centred approach to care. Such an understanding can facilitate a smoother transition and support the sustainable integration of home NIV into daily life. As such, recognising the lived experiences of individuals using home NIV is essential for delivering care that effectively supports long-term adaptation and enhances well-being.

## Figures and Tables

**Table 1:** Participants’ demographics.

Participant’s Pseudonym	Sex	Age Group	Ethnicity	Marital Status	Employment Status	People in the Same Household
Winnie	F	65–99	Chinese	Divorced	Retired	Children
Fatimah	F	65–99	Arab	Widowed	Unemployed	Children
Lily	F	65–99	Chinese	Widowed	Unemployed	Children
Peter	M	45–64	Chinese	Married	Employed	Spouse
Anne	F	45–64	Chinese	Single	Employed	None
Yayah	F	65–99	Malay	Married	Unemployed	Spouse
Victor	M	35–44	Chinese	Married	Employed	Spouse, children
Mary	F	45–64	Chinese	Married	Unemployed	Spouse
Muhammad	M	35–44	Malay	Single	Employed	Relative
Tee	M	65–99	Chinese	Married	Retired	Spouse, children
Yam	F	65–99	Malay	Widowed	Unemployed	Children
Ah Hwee	M	35–44	Chinese	Single	Employed	Relative
William	M	65–99	Chinese	Married	Unemployed	Spouse
Evelyn	F	65–99	Chinese	Married	Unemployed	Spouse
Jennifer	F	45–64	Malay	Widowed	Employed	Relative
Chu	F	35–44	Chinese	Single	Employed	Children
Maria	F	65–99	Chinese	Single	Unemployed	Caregiver
Hapsah	F	65–99	Malay	Married	Unemployed	Children
Charles	M	65–99	Chinese	Married	Unemployed	Children
Khris	M	45–64	Indian	Divorced	Employed	Relative

Legend: F = female, M = male.

**Table 2:** Participants’ clinical information.

Participant’s Pseudonym	Diagnosis	Duration of Home NIV	NIV Mode
Winnie	NMD	More than 1 year	BiPAP
Fatimah	OHS	More than 1 year	BiPAP + daytime oxygen therapy
Lily	OHS	6 months	BiPAP
Peter	OHS	More than 6 months	BiPAP
Anne	OHS	More than 6 months	BiPAP
Yayah	OHS	More than 1 year	BiPAP
Victor	OHS	More than 1 year	BiPAP
Mary	OHS	6 months	BiPAP
Muhammad	OHS	More than 1 year	BiPAP + daytime oxygen therapy
Tee	NMD	More than 1 year	BiPAP + daytime oxygen therapy
Yam	OHS	More than 1 year	BiPAP
Ah Hwee	OHS	More than 1 year	BiPAP
William	OHS	More than 1 year	BiPAP + daytime oxygen therapy
Evelyn	NMD	More than 1 year	BiPAP
Jennifer	OHS	More than 1 year	BiPAP
Chu	OHS	More than 1 year	BiPAP
Maria	NMD	More than 1 year	BiPAP + daytime oxygen therapy
Hapsah	OHS	More than 1 year	BiPAP
Charles	NMD	More than 1 year	BiPAP
Khris	OHS	More than 1 year	BiPAP

Legend: BiPAP = Bilevel Positive Airway Pressure.

**Table 3:** Sub-categories, categories, and themes.

Sub-Categories	Categories	Themes	Quotations
Look forward to using NIV Upset to use NIV Rejected NIV Reconsidered NIV after hospital readmissions	Mixed feelings of excitement and disappointment Remind self that it is part of the treatment	Forced acceptance to use NIV	*“It is something new that you try, you see. Of course, you feel excited.” (Lily)* *“I was disappointed because I (must) use all these things (mask and ventilator) for my whole life. You know, it will be quite a long time. Yes, but then, because of my sickness, I have no choice.” (Jennifer)* *“Sometimes I (was) fed up. I want(ed) to throw (NIV) away. I don’t want to do. Then after a while, I think, no. It’s for my own good.” (Evelyn)*
No more headaches Increased concentration Quality sleep Medical-device-related pressure injury Dry mouth, throat pain Weight loss	Changes in physical Health Changes in mental concentration	Experiencing symptoms relief and side effects	*“When I wake up, I don’t have a headache anymore, and I don’t have that very frustrated feeling when I wake up. You know, it’s easier to wake up with a better mood.” (Anne)* *“After using it, after some time, I really feel that it makes a difference in the ability to concentrate. It lasts longer during the days. It has a significant impact when I reflect (on) it.” (Victor)* *“Every night I must (put) a plaster because of (the) pain. Then, here also must plaster (points to cheeks and chin), here also must plaster, nose.” (Mary)* *“Initially (it) was quite uncomfortable because (the) next morning my throat is very, very dry like having a sore throat, dry throat.” (William)*
Mask re-sizing Mask cleaning Troubleshoot air leaks Purchase mask tubing, mask types, silicone linings	Maintenance of NIV masks Financial expenses of mask accessories	Learning to maintain the NIV mask	*“I frequently see the doctors to find the suitable size. We had the sleep study. They tested the pressure, the mask size, and the strap.” (Muhammad)* *“I used Mama Lemon (dishwashing liquid) to wash every 2 weeks.” (Maria)*
Sleep arrangement NIV placement in bedroom NIV sounds impact family at home	Changes to home environment Family adjusted to NIV sounds	Incorporating NIV into the home environment	*“I put it on the rack-like trolley and then bring the whole thing to the toilet door. Then I go and do (urinate).” (Mary)* *“It’s quite impossible to sleep in the same room, so my mom sleeps in a separate room. Yeah, and I’ll be at the hall for the night. So, I think that’s an arrangement for family if you need to take note of that.” (Tee’s son)* *“I hired a domestic helper. Her job is solely to take care of my mother…She sleeps in the same room.” (Fatimah’s son)*
Stop travelling Continue travelling Family support when travelling	Social adjustment when travelling stops New considerations when travelling with NIV	Readjusting travelling activities	*“When we have to travel, we have to travel with oxygen tanks and everything. So that’s a troublesome part…The machine is big.” (Tee’s son)* *“I’m very worried if I bring (it), I don’t know whether I will damage the machine.” (Anne)* *“When I went to Australia, I brought it…This thing (NIV) is not that heavy.” (Yayah)*
Cannot live without NIV Putting on NIV became easy Anxious when NIV is faulty	Accepted NIV NIV becomes an essential item	NIV as part of their lives	*“It is now my “BFF” (best friend forever). Cannot live without it.” (Jennifer)* *“Without the machine (NIV), I will get breathless when I walk…when the mask has problems, consult the doctor and change for a new one.” (Ah Hwee)*

## Data Availability

The raw data supporting the conclusions of this article will be made available by the authors on request.

## Supplementary Materials

The following supporting information can be downloaded at: https://www.mdpi.com/article/10.3390/nursrep15050176/s1, Thematic analysis.

## Public Involvement Statement

No public involvement in any aspect of this research.

## Guidelines and Standards Statement

This manuscript was drafted against the SRQR (Standards of Reporting Qualitative Research) for qualitative research [47].

## Abbreviations

The following abbreviations are used in this manuscript:

CHRF	Chronic hypercapnic respiratory failure
COPD	Chronic obstructive pulmonary disease
OHS	Obesity hypoventilation syndrome
NMDs	Neuromuscular diseases
NIV	Non-invasive ventilation
HRQoL	Health-related quality of life
BiPAP	Bilevel positive airway pressure
