# Supplementary material for: Adaptation to Long-Term Home Non-Invasive Ventilation for People with Chronic Hypercapnic Respiratory Failure: A Qualitative Study
Source: Nurs Rep. 2025 May 20;15(5):176. doi: 10.3390/nursrep15050176 (PMC12114294; doi:10.3390/nursrep15050176)
Supplement: Supplementary file 1 [file nursrep-15-00176-s001.zip › nursrep-3538434-supplementary.pdf]

SN	Themes	Categories	Sub-categories	Quotations
1.	Forced acceptance to use NIV	Mixed feelings of excitement and disappointment	Upset to use NIV	*“I was disappointed because I (must) use all these things (mask and ventilator) for my whole life. You know, it will be quite a long time. Yes, but then, because of my sickness, I have no choice.” (Jennifer, line 2)*
			Rejected NIV	*" First time the doctor you put on me then I said I don’t want cause I feel I feel very uncomfortable." (Winnie, line 136)*
				“Sometimes I (was) fed up. I want(ed) to throw (NIV) away. I don’t want to do. Then after a while, I think, no. It’s for my own good.” (Evelyn, line 90-92)
			Look forward to using NIV	*“It is something new that you try, you see. Of course, you feel excited.” (Lily, line 8)*
		Remind self that it is part of the treatment	Reconsidered NIV after hospital readmissions	*"Yah got problem ah. Few months I must go inside the hospital." (Peter, line 59)*
				"there was a situation where he was very weak and very easily tired and all. So so eventually led him to umm coming into A&E (Accident and Emergency) and going for ICU (Intensive Care Unit) for almost about two weeks on ICU in the ICU, close to, so after that when he, because of all of the, you know, intubation and everything" (Tee's son, line 3)
2.	Experiencing symptom reliefs and side effects	Changes in physical health	Medical device-related pressure injury	“Every night I must (put) a plaster because of (the) pain. Then, here also must plaster (points to cheeks and chin), here also must plaster, nose.” (Mary, line 15)
			Dry mouth, throat pain	*“Initially (it) was quite uncomfortable because (the) next morning my throat is very, very dry like having a sore throat, dry throat.” (William, line 2)*
			Weight loss	*"My weight. From one hundred and twenty kilos, I went down to ninety-four kilos." (Yayah, line 9)*

		Changes in mental concentration	No more headaches	*“When I wake up, I don’t have a headache anymore, and I don’t have that very frustrated feeling when I wake up. You know, it’s easier to wake up with a better mood.” (Anne, line 4-6)*
				"So when I was hospitalised for nine days, the doctor did all sorts of things, use this use that. That was when my years of experiencing headache instantly disappeared." (Yayah, line 1)
			Increased concentration	*“After using it, after some time, I really feel that it makes a difference in the ability to concentrate. It lasts longer during the days. It has a significant impact when I reflect (on) it.” (Victor, line 4)*
				"Only at the end of the day you know you feel tired, but you will not lose focus" (Anne, line 44)
			Quality sleep	*"So now when I drive right, I no longer feel sleepy." (Anne,line 48)*
				"I can sleep soundly and wake up feeling fresh. Before using this, whenever I sat down, I slept." (Yayah, line 5)
3.	Learning to maintain the NIV mask	Maintenance of NIV masks	Mask re-sizing	*“I frequently see the doctors to find the suitable size. We had the sleep study. They tested the pressure, the mask size, and the strap.” (Muhammad, line 7)*
			Mask cleaning	*"I used Mama Lemon (dishwashing liquid) to wash every 2 weeks.” (Maria, line 16)*
				"Then I clean, first mask I don’t know, one day I take out and wash. Then after some time then the whole mask come out so I think maybe because really cannot use water can use swab." (Winnie, line 159-167)
			Troubleshoot air leaks	*"At night. I will be at the hall. So in his room, basically hmm I half shut the door. So at least the sound won't be so loud, then basically (I'm) at the hall if anything, I mean sometimes touch wood there might be leaks, collapse, leads give away." (Tee's son, line 102-105)*

				"The (other) two ah leaking and leaking the whole night. Very, once leaking the machine will have noise you know. Beep beep beep beep very noisy." (Mary, line 44)
		Financial expenses of mask accessories	Purchase mask tubing, mask types, silicone linings	*"Uh the other thing is more towards that, you know, in the perspective of the the the wear and tear, uh it seems to be quite, quite uh... It seems to be uh first thing is that it seems to tear quite easily, The the lining of the the the silicon lining itself seems to crack, tear quite easily. When I change the memory foam uh I don't really have that issue, but I can also realize that the head gear and the strap itself also you know, it lasts me about two to three months because of the pressure, the pressure that I need to put on." (Victor, line 10-12).*
4.	Incorporating NIV into the home environment	Changes to home environment	Sleep arrangement	*"So what happened is um the the the arrangement um is that um I would usually be in the hall in the night. So I mean, because we are the caregiver, I mean caregiver so my mom daytime, my mom will basically manage, but at night. I will be at the hall."(Tee's son, line 102)*
				"It's quite impossible to sleep in the same room, so my mom sleeps in a separate room. Yeah, and I'll be at the hall for the night. So, I think that's an arrangement for family if you need to take note of that." (Tee's son, line 107)
				"I hired a domestic helper. Her job is solely to take care of my mother…She sleeps in the same room." (Fatimah's son, line 165)
			NIV placement in bedroom	*"It's it's pretty smalll. Hahaha. You can fit in one bag and take out because I I was I was renting a place before I found my my found a new home lah so the the house basically, lantai parket (parquet flooring in Malay) then I just put near to the plug and just sleep beside the plug." (Muhammad, line 20)*
				"I put it on the rack-like trolley and then bring the whole thing to the toilet door. Then I go and do (urinate)." (Mary, line 50)

		Family adjusted to NIV sounds	NIV impacts family at home	*"So what happened is um the the the arrangement um is that um I would usually be in the hall in the night. So I mean, because we are the caregiver, I mean caregiver so my mom daytime, my mom will basically manage, but at night. I will be at the hall."(Tee's son, line 102)*
				"It's quite impossible to sleep in the same room, so my mom sleeps in a separate room. Yeah, and I'll be at the hall for the night. So, I think that's an arrangement for family if you need to take note of that." (Tee's son, line 107)
5.	Readjusting travelling activities	Social adjustment when travelling stops	Stop travelling	*my daughter wants me to go overseas holiday. Yeah I say cannot lah have to bring the mask along. So problem you see...As in you travel ah, you change change hotel I mean all this har, you have to carry what lorh, I think that's the problem...You know you have to take out and keep you know. That's the problem...Since I have this mask I never go anywhere. Yeah, even I last time I used to go market all this. Now I also my children do all this." (Winnie, line 68-73)*
		New considerations when travelling with NIV	Continue travelling	*"When I went to Australia, I brought it…This thing (NIV) is not that heavy." (Yayah, line 22)*
				"Maybe make the trip shorter, you know. Don't go for two weeks...OK. But when it comes to the machine part maybe make it a shorter trip." (Anne, line 26-28)
			Family support when travelling	*"When we have to travel, we have to travel with oxygen tanks and everything. So that's a troublesome part…The machine is big." (Tee's son, line 44)*

6.	NIV as part of their lives	Accepted NIV	Cannot live without NIV	*“It is now my “BFF” (best friend forever). Cannot live without it.” (Jennifer, line 89)*
			Putting on NIV became easy	*"Hospitalization of one week they actually try to put me on the mask every night. So eventually I accepted it. OK. And then now I will automatically put it on because very simple." (Anne, line 5)*
		NIV becomes an essential item	Anxious when NIV is faulty	*“Without the machine (NIV), I will get breathless when I walk…when the mask has problems, consult the doctor and change for a new one.” (Ah Hwee, line 47)*
